# Resignation and Reorganization

**Published:** 1878-07

**Authors:** 


### RESIGNATION AND REORGANIZATION.

Within a few weeks after the close of the last session of the Ohio College of Dental Surgery, all the members of the Faculty resigned their positions as teachers in that institution. This was done in order that the Trustees might effect a reorganization, and make some changes that they deemed important.

The reorganization has been made, in which three members of the former faculty have been reappointed, viz: Dr. Wm. Clendenin, to the chair of Anatomy, Dr. J. L. Cilley, to the chair of Physiology, and Dr. J. S. Cassidy, to Chemistry, and Materia Medica. Dr. A. O. Rawls, of Lexington, Ky., was assigned to the chair of Pathology and Therapeutics instead of Dr. F. Brunning; Dr. James Taylor to Operative Dentistry instead of Dr. J. Taft; Dr. H. A. Smith to Clinical Dentistry instead of Dr. H. M. Reid, and Dr. J. L. Clayton, of Shelbyville, Ind., to Mechanical Dentistry instead of Dr. N. S. Hoff.

In addition to these, quite a number of clinical lecatureres and operators have been appointed. The members of the faculty as here indicated—the announcement has not yet made its appearance—are all well known to the profession, and except two of them having occupied the position of teachers before.

These gentlemen know well the needs of the institution, and we are confident are disposed to put forth the effort and energy requisite to make it a success. We trust that in this respect a new era has dawned. Favorable circumstances now exist which have been wanting hitherto.

The college is free from debt and has some funds on hand, and is released from taxation, and the running expenses can now be made less than ever before.

The announcement will be issued in a few days. We earnestly ask for this old and honored institution the hearty aid and co-operation of the profession of the west.

